# Redirection of CD4+ and CD8+ T lymphocytes via an anti-CD3 × anti-CD19 bi-specific antibody combined with cytosine arabinoside and the efficient lysis of patient-derived B-ALL cells

**DOI:** 10.1186/s13045-015-0205-6

**Published:** 2015-10-06

**Authors:** Dongmei Fan, Wei Li, Yuqi Yang, Xiaolong Zhang, Qing Zhang, Yan Yan, Ming Yang, Jianxiang Wang, Dongsheng Xiong

**Affiliations:** State Key Laboratory of Experimental Hematology, Institute of Hematology and Hospital of Blood Diseases, Chinese Academy of Medical Sciences and Peking Union Medical College, Tianjin, 300020 People’s Republic of China; Department of Maxillofacial and E.N.T. Oncology, Tianjin Medical University Cancer Institute and Hospital, National Clinical Research Center of Cancer, Key Laboratory of Cancer Prevention and Therapy, Tianjin, 300060 People’s Republic of China; School of Pharmacy, Tianjin Medical University, Tianjin, 300070 People’s Republic of China

**Keywords:** Bi-specific antibody, Diabody Anti-CD3, Anti-CD19, Disulfide crosslinks, B-ALL, Cancer immunotherapy

## Abstract

**Background:**

B-acute lymphoblastic leukemia (B-ALL) is derived from B cell progenitors. Recently, the development of appropriate combinations of chemotherapy and immunotherapy represents a promising approach for eliminating cancer. We previously constructed an anti-CD3 × anti-CD19 bi-specific antibody in a diabody configuration and its disulfide-stabilized format (ds-diabody). The combination of the diabody or ds-diabody and Ara-C was highly effective in enhancing the cytotoxicity of T cells against the CD19+ human leukemia cell-line, Nalm-6, both in vitro and in vivo. This study verified whether B-ALL patient-derived cells were sensitive to the diabody or ds-diabody and low-dosage Ara-C combination.

**Methods:**

This study aimed to detect the B7 family members B7.1 (CD80) and B7.2 (CD86) that were expressed in B-ALL patient-derived cells pre-treated by Ara-C (0.25 μM) and to determine the targeted killing ability of T cell subtypes induced by the diabody or ds-diabody combination with Ara-C both in vitro and in vivo. We also determined the levels of the cytokines that were released by activated CD4+ or CD8+ T cells during therapy.

**Result:**

Low-dose Ara-C enhanced CD80 and CD86 expression in nearly 50 % of specimens of B-ALL patient-derived cells. A combination of diabody or ds-diabody and Ara-C enhanced T cell against B-ALL cells in vitro and in vivo. Both CD8+ and CD4+ T cells were potently activated. Expression of CD25 and CD69 was augmented equally by CD4+ or CD8+ T cells. However, CD8+ T cells made the major contribution by redirecting target cell lysis in a granzyme B and perforin-dependent mechanism. CD4+ T cells played an important immunomodulatory role by secreting IL2. Consequently, IL3, IL6, TNFα, and IFNγ were also released by CD4+ or CD8+ T cells following diabody-mediated T cell activation.

**Conclusion:**

T cell therapy induced by diabody or ds-diabody combined with low dose of Ara-C was effective against cancer cell-lines and in clinical trials. In vivo, the ds-diabody was more efficient than its parent diabody due to its enhanced stability.

## Introduction

B-lymphoblastic leukemia/lymphoma, also known as B-acute lymphoblastic leukemia (B-ALL), is a hematological malignancy that is derived from B cell progenitors. B-ALL occurs at any age but is predominantly seen in children [1, 2]. Risk-adapted intensive chemotherapy is effective in treating most children with B-ALL, but this approach is less successful in adults [3]. Chemotherapy is traditionally considered to kill cancer cells via direct cytotoxicity. Therefore, these drugs often generate severe side effects in patients such as nausea, vomiting, bone marrow depression, and alopecia. A common cause of treatment failure includes acquired resistance of tumor cells to conventional chemotherapy [4]. Targeted therapy represents a promising alternative to conventional chemotherapy in various types of cancer [5–8]. Monoclonal antibodies have played a major role in lymphoma therapy for more than a decade [9–11]. A series of monoclonal antibodies (mAbs) that specifically target different tumor cell surface antigens have been tested in both experimental and clinical studies [12, 13]. The anti-CD20 mAb, rituximab, has substantially improved clinical outcomes in Burkitt’s lymphoma/leukemia and is currently applied in de novo B-precursor ALL [14]. The MoAbs that are directed against CD22, linked to cytotoxic agents including, either to calicheamicin (i.e., inotuzumab ozogamicin) or bacterial toxins (e.g., epratuzumab) are explored in refractory/relapsed childhood and adult ALL [15]. A novel anti-CD19 monoclonal antibody, GBR 401, exerts a potent in vitro and in vivo cytotoxic activity against primary samples from patients representing various B cell malignancies [16]. In addition, novel immunotherapeutics targeting B cell receptor signaling (e.g., ibrutinib) [17], T cell receptor (e.g., CART19) [18], and NK cells (e.g., AFM13) [19] are being developed.

In addition to the development of therapeutics for new targets, another approach to improve current antibody therapy is the development of bi-specific antibodies (bsAbs). In principle, bsAbs are generated by the fusion of the minimal binding domains (fragment variable) of two mAbs via flexible peptide linkers. By simultaneous binding to the activating CD3 complex and a tumor-associated surface antigen (TAA), they can trigger efficient T cell-mediated tumor cell lysis in a TCR- and MHC-independent manner [20–24]. Various formats of bsAbs engineered to redirect cytotoxic T cells (CTLs) have demonstrated therapeutic efficacy in preclinical and clinical settings by binding to tumor-associated antigens. The potential of this mode of therapy was demonstrated pre-clinically in hematopoietic cancers [25, 26]. For example, the CD19 × CD3 BiTE (blinatumomab or MT103) has been studied in phase II trials for B-ALL [27].

Moreover, some studies have shown that chemotherapy might also enhance the immune response of the host against the tumor itself [28]. As mounting evidence suggests that the therapeutic efficacy of some chemotherapeutic drugs relies on their capability to interact with the immune system, the development of appropriate combinations of chemotherapy and immunotherapy represents a promising approach for eliminating cancer cells [29, 30].

Recently, we constructed a novel bi-specific anti-CD3 × anti-CD19 diabody and its disulfide-stabilized format (ds-diabody) [31]. The cytotoxicity of these two diabodies showed no dramatic differences in vitro. However, in vivo, the ds-diabody was more efficient than its parental diabody in inducing tumor cell lysis because of its increased stability [31]. Osada et al*.* used chemotherapy to sensitize tumor targets to cytotoxicity mediated by bi-specific antibodies that were directed to T cells [32]. Tretter reported that taxanes could sensitize BiAb killing [33].

In the present study, Ara-C up-regulated CD80 expression on the CD19+ human leukemia cell-line Nalm-6. A combination of the diabody plus Ara-C induced greater CTL activity against Nalm-6 cells both in vitro and in vivo [34]. Ara-C, which is one component of the most widely used regimens for treating ALL, was used in this study at a low dose.

This study aimed to verify whether B-ALL patient-derived cells were also sensitive to combined treatment with the diabody or ds-diabody and low-dose Ara-C. The purpose of the study was to detect the B7 family members B7.1 (CD80) and B7.2 (CD86) that were expressed in B-ALL patient-derived cells following pre-treatment with Ara-C and to determine whether the combination of the diabody or ds-diabody with Ara-C enhanced the capacity of sub-populations of T cells to kill the tumor cells more effectively in vitro and in vivo.

## Results

### Co-stimulation of molecular expression on B-ALL cells

Among the 21 samples of B-ALL cells, CD80 and CD86 expression increased 100 % in 10 of 21 samples following treatment with Ara-C (Table 1, patient no. 1, 4, 5, 6, 9, 13, 15, 16, 20, 21). The samples in which CD80 or CD86 increased over 100 % were chosen for the following experiments. The results are expressed as the average of the selected 10 samples.

**Table 1:** Co-stimulation of molecular expression on B-ALL cells (%)

Number	Patient ID	CD80	CD80 (Ara-C)	CD86	CD86 (Ara-C)
1	149290	5.48	12.84	5.20	12.49
2	152618	4.56	5.30	5.81	7.21
3	143674	8.71	9.55	41.77	59.73
4	146937	2.69	10.30	10.45	20.39
5	137930	4.98	13.86	4.37	31.86
6	153776	4.15	9.55	4.25	16.41
7	153832	7.21	7.17	24.05	27.81
8	154438	2.34	2.75	1.72	3.11
9	154915	3.94	8.27	4.32	15.12
10	161080	0.61	1.04	3.01	5.12
11	164302	1.02	1.32	0.31	0.44
12	151732	0.81	1.32	16.51	17.55
13	161015	3.73	33.65	16.02	47.75
14	160483	9.96	10.86	2.93	3.45
15	161101	5.27	5.73	17.53	43.26
16	141322	5.25	12.90	6.60	9.07
17	178545	3.01	4.41	3.97	6.04
18	172540	9.87	10.1	11.70	13.66
19	173869	3.68	3.38	9.48	11.95
20	175981	4.57	5.65	0.42	1.65
21	153971	4.48	17.44	0.44	24.83

### Cytotoxicity mediated by the diabody or ds-diabody in vitro

**Fig. 1:**
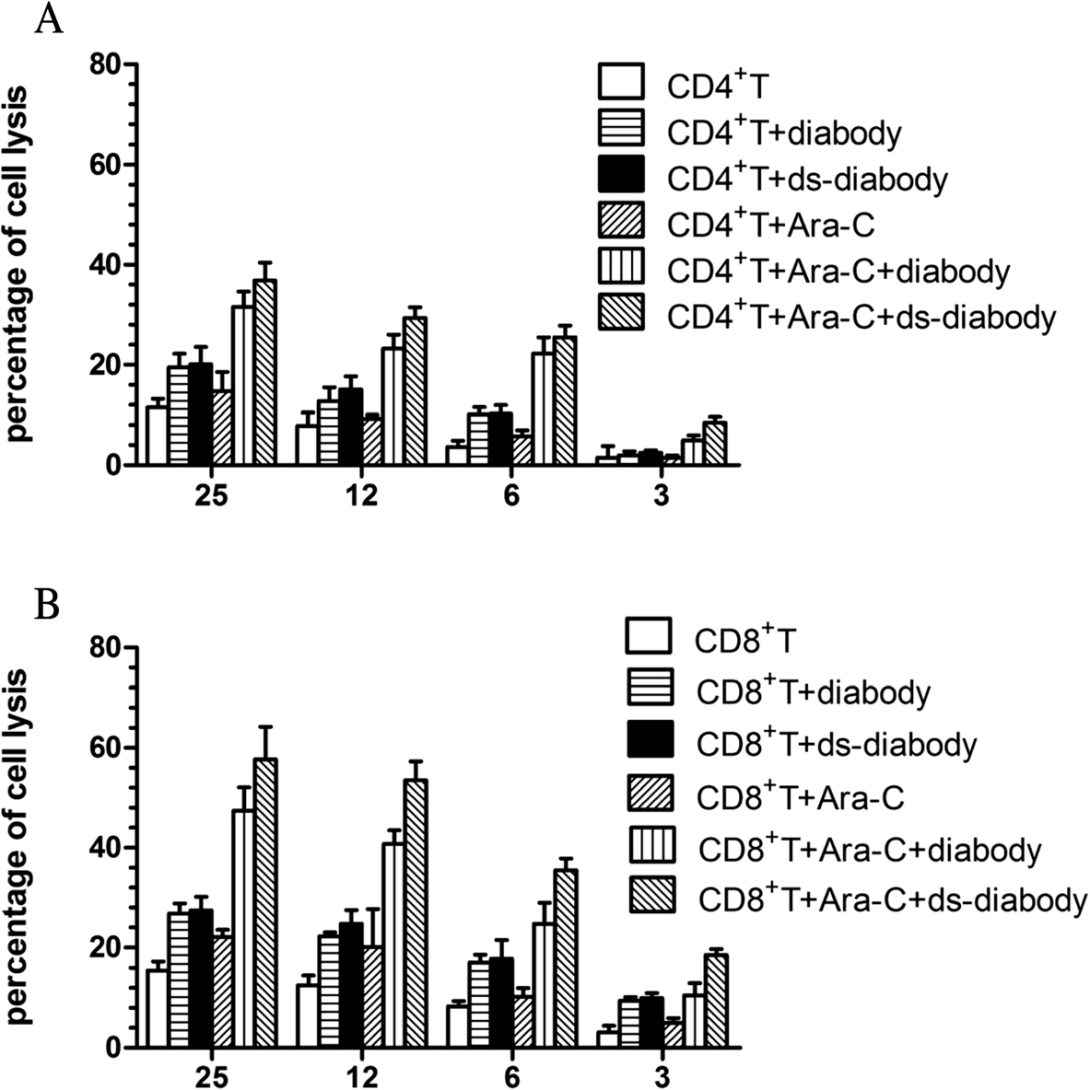
Cytotoxicity of human T cell subclone in different E/T ratios mediated by diabody or ds-diabody (1.0 pM) in a non-radioactive cytotoxicity assay. **a** Cytotoxicity of CD4+ T cells. **b** Cytotoxicity of CD8+ T cells. Effector-to-target cell ratio ranged from 25:1 to 3:1. The killing effect of activated CD4+ or CD8+ T cells reached the maximum when Ara-C (0.25 μM) and the diabody or ds-diabody were both added. There was no statistical difference between the parent diabody and ds-diabody in mediating the lysis of tumor cells. The killing effect of CD8+ T cells was greater than that of CD4+ T cells. Data shown are the means ± SD of experiments for each B-ALL sample cells performed in quadruplicate

Cytotoxic effects were enhanced along with increasing the ratio of the effector cells to the target cells. In the presence of diabody or ds-diabody, the cytotoxic effect was obviously strengthened. If the tumor cells were stimulated by pre-treatment with low doses of Ara-C before, the killing effect of activated T cells also increased and reached the maximum when the diabody or ds-diabody was added. The ds-diabody was as efficacious as the parent diabody. There was no statistical difference between the parent diabody and ds-diabody in mediating the lysis of tumor cells. Without doubt, the killing effects of CD8+ T cells were greater than CD4+ T cells. However, CD4+ T cells contributed to the cytotoxic effects (Fig. 1).

### Up-regulation of activation markers by activated T cells

**Fig. 2:**
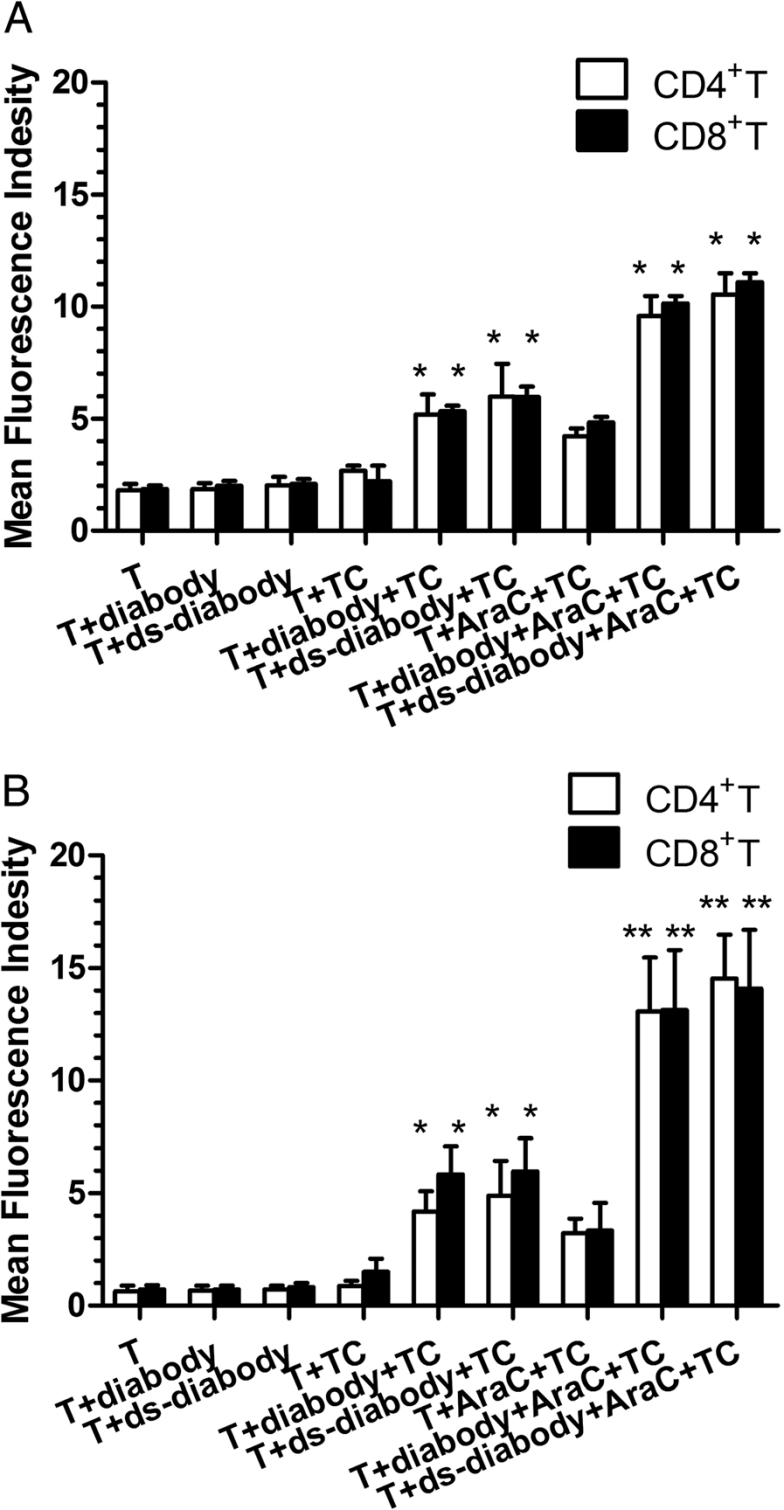
Activation markers expressed by activated T lymphocyte subclones. Expressions of T cell activation markers CD25 (**a**) and CD69 (**b**) were significantly increased when T cells were incubated with target cells and diabody. The T cell activation markers increased to higher levels when T cells were incubated with the tumor cells stimulated by Ara-C, then the tumor cells themselves. Expressions of CD25 and CD69 increased equally in both CD4+ and CD8+ T cells, *p < 0.05, **p < 0.01. *TC* target cells

The T cells alone or T cells were incubated with diabody or ds-diabody were set up as control. Expression of T cell activation markers CD25 and CD69 obviously increased in CD4+ or CD8+ T cells incubated with target cells and the diabody (MFI: CD4+ CD25+: 5.19 ± 0.91, CD4+ CD69+: 4.18 ± 0.90, CD8+ CD25+: 5.31 ± 0.30, CD8+ CD69+: 5.84 ± 1.28) or ds-diabody (10 pM) (MFI: CD4+ CD25+: 5.99 ± 1.45, CD4+ CD69+: 4.89 ± 1.55, CD8+ CD25+: 5.97 ± 0.47, CD8+ CD69+: 5.56 ± 1.06). Furthermore, the T cell activation markers increased much more when CD4+ or CD8+ T cells were incubated with the tumor cells stimulated by Ara-C than with the tumor cells itself, especially in the presence of diabody (MFI: CD4+ CD25+: 9.57 ± 0.90, CD4+ CD69+: 13.07 ± 2.40, CD8+ CD25+: 10.14 ± 0.33, CD8+ CD69+: 13.15 ± 2.65) or ds-diabody (MFI: CD4+ CD25+: 10.54 ± 0.95, CD4+ CD69+: 14.52 ± 1.97, CD8+ CD25+: 11.09 ± 0.39, CD8+ CD69+: 14.11 ± 2.59) (Fig. 2).

### Expressions of perforin and granzyme B in the activated T cell subpopulation

**Fig. 3:**
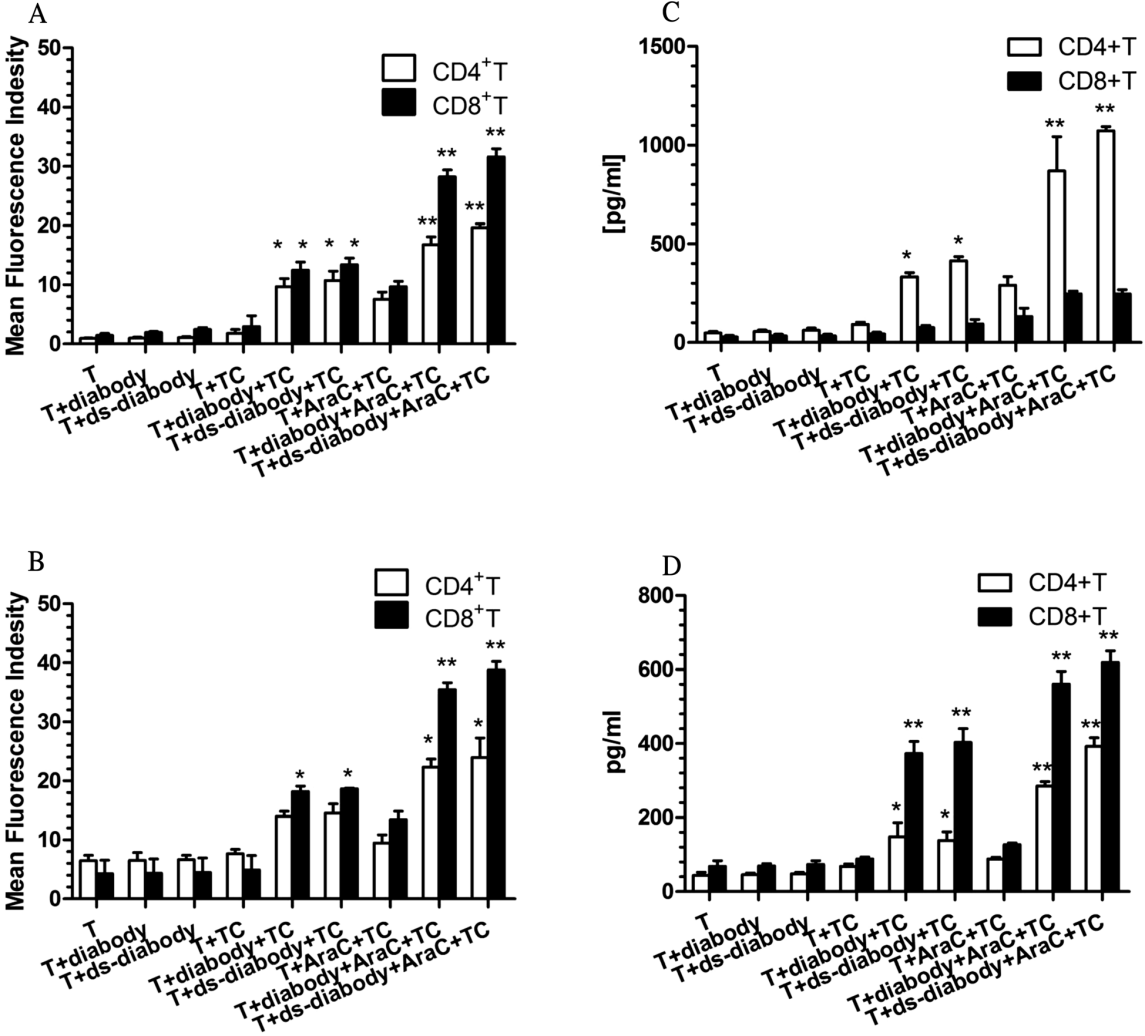
Expressions of perforin, granzyme B, IL2 and IL6 by activated T cell subpopulation. There was a greater percentage of perforin/granzyme B/IL2/IL6 CD4+ or CD8+ T cells after co-culturing tumors, T cells, and diabody or ds-diabody compared to the control. Tumor cells pre-incubated with Ara-C stimulated more perforin (**a**)/granzyme B (**b**)/IL2 (**c**)/IL6 (**d**)expressed by T subpopulation cells than tumor cells alone. Moreover, CD8^+^ T cells released more perforin, granzyme B, and IL6 than CD4^+^ T cells, and CD4+ T cells released more IL2 than CD8+ T cells. *p < 0.05 and **p < 0.01 were compared to the controls. T subpopulation groups were the controls. *TC* target cells

It is well known that T cells kill tumors by the perforin/granzyme B pathways. We observed a greater percentage of perforin/granzyme B-expressing T cells after co-culturing tumors, T cells, and diabody compared to the control. Furthermore, tumor cells pre-incubated with Ara-C stimulated more perforin (MFI: CD8+: 28.24 ± 1.18, CD4+: 16.77 ± 1.35) and granzyme B (MFI: CD8+: 35.47 ± 1.20, CD4+: 22.30 ± 0.40) than tumor cells alone. As expected, activated CD8+ T cells expressed much more perforin/granzyme B than CD4+ T cells. The expressions of perforin/granzyme B between the diabody and ds-diabody groups had no obvious difference (Fig. 3a, b).

### IL2 and IL6 released by activated T cell subpopulation

IL2 that was produced by the CD4+ T cells alone (the value was 48.7 ± 7.3 pg/ml) significantly increased when CD4+ T cells were incubated with tumor cells and the diabody ( 333.0 ± 22.5 pg/ml). Moreover, tumor cells stimulated by Ara-C induced CD4+ T cells to produce more IL2 (290.6 ± 33.5 pg/ml) than tumor cells alone (*p* < 0.01). When tumor cells stimulated by Ara-C and diabody were added, IL2 released by T cells was much more higher (869.1 ± 134.6 pg/ml). Although CD8+ T cells released secretion of IL2, no obvious differences were observed. The result of the ds-diabody group was similar to that found with the diabody (Fig. 3c). IL6 was mainly released by CD8+ T cells (Fig. 3d). In the presence of diabody or ds-diabody, tumor cells stimulated by Ara-C induced CD8+ T cells to produce the highest amount of IL6 (diabody: 578.7 ± 52.5 pg/ml, ds-diabody: 618.8 ± 31.3 pg/ml). They are much higher than that of CD8+ T cells without combining diabody/ds-diabody (127.0 ± 4.8 pg/ml) or Ara-C (88.57 ± 5.3 pg/ml) (*p* < 0.01).

### IL3, TNFα, and IFNγ released by activated T cell subpopulation

**Fig. 4:**
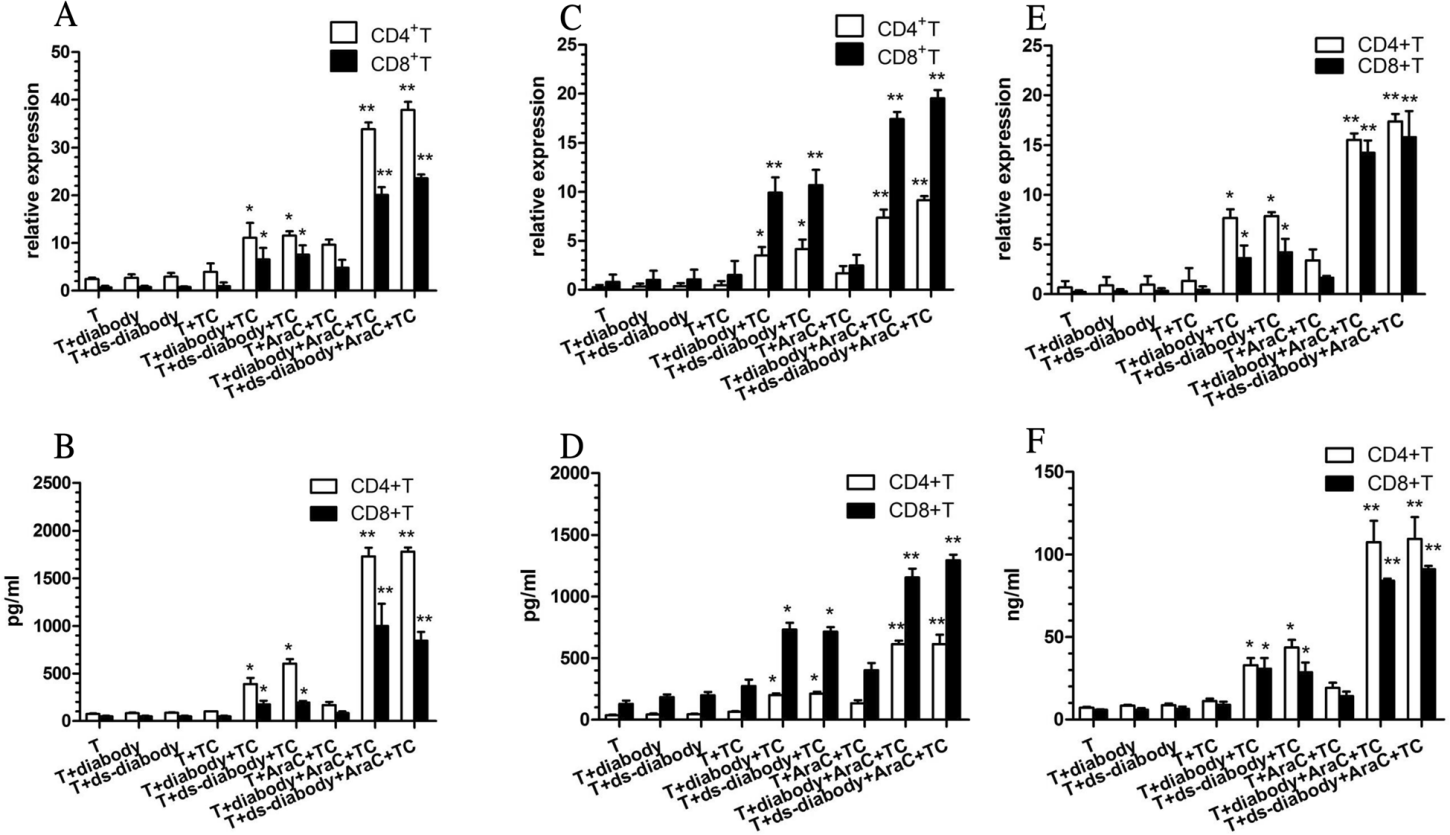
IL3, TNFα, and IFNγ released by activated T cell subpopulation. CD4+ or CD8+ T cells incubated with tumor target cells and the diabody or ds-diabody (10 pM) released higher amounts of IL3 (**a**, **b**), TNFα (**c**, **d**), and IFNγ (**e**, **f**). Tumor cells incubated with Ara-C stimulated CD4+ or CD8+ T cells, releasing the highest IL3, TNFα, and IFNγ amounts in the presence of diabody or ds-diabody. CD4+ T cells released more IL3 than CD8+ T cells, while CD8+ T cells produced more TNFα. IFNγ had no difference when released by both T cell subgroups. *p < 0.05 and **p < 0.01 were compared to the controls. T subpopulation groups were the controls. *TC* target cells. qPCR:a,c,e.ELISA:b,d,f

The T cell subgroups were used as a control. CD4+ or CD8+ T cells incubated with tumor target cells and the diabody (10 pM) released higher amounts of IL3 (CD4+ T: 388.30 ± 44.11 pg/ml, CD8+ T: 175.85 ± 28.37 pg/ml), TNFα (CD4+ T: 201.32 ± 13.22 pg/ml, CD8+ T: 732.26 ± 45.90 pg/ml), and IFNγ (CD4+ T: 32.95 ± 4.39 ng/ml, CD8+ T: 30.85 ± 6.49 ng/ml). Also, tumor cells incubated with Ara-C stimulated CD4+ or CD8+ T cells, releasing the highest IL3 (CD4+ T: 1729.28 ± 92.46 pg/ml, CD8+ T: 999.39 ± 133.09 pg/ml), TNFα (CD4+ T: 613.45 ± 28.26 pg/ml, CD8+ T: 1155.64 ± 72.15 pg/ml), and IFNγ (CD4+ T: 107.60 ± 12.69 ng/ml, CD8+ T: 84.21 ± 1.23 ng/ml) in the presence of diabody. CD4+ T cells released more IL3 than CD8+ T cells, while CD8+ T cells produced more TNFα. IFNγ had no difference when released by both T cell subgroups. Ds-diabody also mediated the CD4+ or CD8+ T cells to release as many of these cytokines as diabody (Fig. 4b,d,f). The results of qPCR were correspondence to ELISA(Fig.4a,c,e).

### Tumor growth inhibition in vivo

The antitumor activity of the diabody or ds-diabody with or without Ara-C and that of the control groups is shown in Table 2. Five B-ALL sample cells were successfully established in the tumor engraftment models on NOD/SCID mice. No obvious tumor-inhibiting effect was observed in the mice treated with T cells, diabody, ds-diabody, or Ara-C alone. Also, T cells combined with Ara-C did not display apparent antitumor activity. The mice treated by T cells and diabody or ds-diabody (*p* < 0.05) showed obvious antitumor activity. Furthermore, when Ara-C was added, the tumor inhibition of the combination was more significant than that of the other groups (*p* < 0.01).

**Table 2:** Tumor growth in vivo (%)

	Patient 1		Patient 2		Patient 3		Patient 4		Patient 5		Average	
	CD45+	CD19+	CD45+	CD19+	CD45+	CD19+	CD45+	CD19+	CD45+	CD19+	CD45+	CD19+
PBS	87.8 ± 6.8	87.6 ± 9.9	83.5 ± 7.3	83.9 ± 8.9	79.2 ± 9.2	79.6 ± 6.7	85.1 ± 6.1	85.7 ± 8.3	88.3 ± 8.1	88.5 ± 10.4	84.8 ± 5.6	85.1 ± 5.5
T	82.1 ± 5.9	82.8 ± 6.1	80.1 ± 9.4	80.6 ± 5.4	78.2 ± 7.1	78.9 ± 7.8	81.2 ± 5.9	81.0 ± 7.9	80.9 ± 10.9	81.2 ± 7.8	80.5 ± 2.3	80.9 ± 2.0
T + diabody	60.0 ± 7.2	59.8 ± 5.7	60.1 ± 1.6	59.5 ± 5.6	58.8 ± 6.1	58.5 ± 6.5	62.5 ± 2.6	61.9 ± 6.4	62.6 ± 5.7	63.0 ± 3.4	60.8 ± 2.0*	60.5 ± 2.0*
T + ds-diabody	49.5 ± 5.5	48.7 ± 6.2	47.6 ± 3.2	48.1 ± 7.1	46.3 ± 4.6	46.0 ± 5.7	49.1 ± 4.3	49.7 ± 5.1	50.4 ± 7.2	50.7 ± 2.7	48.6 ± 2.3*	48.6 ± 2.6*
T + Ara-C	77.0 ± 8.1	77.8 ± 7.4	67.3 ± 3.3	68.0 ± 4.6	70.1 ± 5.6	69.9 ± 6.9	72.0 ± 6.8	70.8 ± 3.8	75.1 ± 5.3	75.5 ± 5.5	72.3 ± 5.0	72.4 ± 2.5
T + Ara-C + diabody	21.5 ± 3.2	20.8 ± 2.8	15.8 ± 3.1	15.0 ± 3.3	14.3 ± 2.7	13.9 ± 3.7	19.7 ± 2.1	19.1 ± 0.9	17.8 ± 1.1	17.9 ± 2.7	17.8 ± 3.5**	17.3 ± 3.4**
T + Ara-C + ds-diabody	1.3 ± 0.5	1.2 ± 0.4	0.8 ± 0.2	0.7 ± 0.1	0.7 ± 0.2	0.5 ± 0.1	2.5 ± 0.3	2.4 ± 0.4	2.0 ± 1.2	2.1 ± 0.5	1.5 ± 0.8**	1.4 ± 0.9**

*p < 0.05; **p < 0.01

## Discussion

Traditional chemotherapy disrupts fundamental regulatory pathways essential for tumor cell growth and survival. Meanwhile, they usually cause severe toxicity to normal tissues and induce drug resistance. Many standard and high-dose chemotherapy regimens are immunosuppressive, contributing to leukopenia and lymphopenia [35]. However, an abundance of evidence suggests that low-dose chemotherapy may augment tumor immunity via a variety of pathways. The critical pathway is one in which chemotherapy can adjust the tumor microenvironment by modulating the expression of tumor antigens, accessory molecules of T cell activation or inhibition, and molecules involved in antigen processing and presentation [36].

In the previous study, we found that Ara-C up-regulated CD80 expressed on CD19+ human leukemia cell-line Nalm-6 and some specimens of B-ALL patient-derived cells. The CD80–CD28 signaling pathway was the most potent co-stimulatory pathway facilitating antigen-specific T cell activation. Here, we found that CD80 and CD86 expression increased nearly 100 % on 50 % of B-ALL samples after being treated with Ara-C. An assay of human acute myelocytic leukemia (AML) cells demonstrated that exposure to doses of Ara-C as low as 0.05 mM led to a marked increase of CD86 and a lesser increase of CD80 [37]. Most specimens showed enhanced expressions of CD80 or CD86, but some subtypes of AML, especially FAB M0 and M1, showed no modification or a decrease of expression. Thus, different drugs may stimulate different subtypes of malignant cells through a variety of signaling pathways.

Peripheral CTLs with specificity against leukemic antigens found in chronic myeloid leukemia (CML) patients and healthy individuals indicated that a natural anti-leukemic immune response might exist in vivo [38, 39]. Additionally, mouse leukemic cells exposed in vivo to Ara-C were more susceptible to CTL-mediated cell killing [36]. This result suggested that the specific anti-leukemic activity of CTL, acting in synergy with enhanced sensitivity of leukemic cells to immune-mediated cell killing induced by chemotherapy, might eradicate minimal residual disease [40]. According to our previous study, cytotoxicity would be enhanced if CTL were recruited around the tumor cells. Furthermore, in the presence of B-ALL samples, both CD8+ and CD4+ T cells were potently activated. Expression of CD25 and CD69 was up-regulated equally in CD4+ T cells and CD8+ T cells. However, CD8+ T cells made the major contribution to redirect target cell lysis by functional expression of granzyme B and perforin. CD4+ T cells also participated in the perforin/granzyme pathway to lyse the target cells. However, CD4+ T cells played a main role in modulating other immunocyte functions including CD8+ T cells by releasing IL2. Previous studies have indicated that the cytokine secretion profile differs between CD4+ and CD8+ cells. The CD8+ cells have been shown to secrete Th1 cytokines, whereas CD4+ cells secrete both Th1 and Th2 cytokines [41]. Originally, it was suggested that a Th1-type response leads to tumor regression, whereas Th2 cytokines down-regulate antitumor immunity [42]. In normal conditions, Th1 and Th2 cytokines secreted by immunocytes modulate the balance of immunological response to tumor growth. Thus, we speculated that in the tumor microenvironment around the target cells in vivo, diabody or ds-diabody and Ara-C would enhance antitumor response and impair immunosuppression.

The use of bi-specific antibodies essentially leads to strong T cell activation; in the meantime, they also induce the T cells to produce plenty of proinflammatory cytokines [43], such therapies might also trigger tumor cells to employ immunosuppressive strategies to escape antibody-mediated tumor cell lysis. Moreover, reduced T cell activation and impaired tumor cell lysis were observed in some patient samples [44, 45]. As a consequence of bi-specific antibody-mediated T cell activation in this paper, IL3, TNFα, and IFNγ were released into cell culture supernatants either by CD4+ or CD8+ T cells. Among them, IL3 was identified among the most important cytokines for regulating mast cell growth and the differentiation, migration, and effector function activities of many hematopoietic cells [46]. It is predominantly produced by activated T cells, natural killer (NK) cells, and mast cells. IL3 causes severe hypersensitivity reactions and plays a pivotal role in exacerbating inflammatory responses in vivo. This also might help overcome the immunosuppressive tumor milieu and reactivate pre-existing, tumor-specific T lymphocytes within the scope of an antigen-specific immunotherapy.

The most common side effect associated with the bi-specific immunotherapy is cytokine release syndrome (CRS), an inflammatory symptom resulting from cytokine elevations associated with T cell engagement and proliferation [47]. The most significantly elevated cytokines in the CRS are IL10, IL6, and IFNγ [48]. IL6 is one of the most important cytokines involved in CRS. We measured cytokine IL6 by ELISA. The quantity of IL6 released in the supernatant is much lower than other cytokines. We just measure it in vitro with limited time of incubation. However, we did not measure IL6 in vivo, but the mice during the period of treatment have no obvious abnormal behaviors as anorexia, weight loss, allergy, and so on. So, the exact quantity of release of IL6 associated with T cell engagement by diabody in vivo needs to be confirmed in the future.

T cell activation and dysfunction relies on direct and modulated receptors. Based on their functional outcome, co-signaling molecules can be divided as co-stimulators and co-inhibitors, which positively and negatively control the priming, growth, differentiation, and functional maturation of a T cell response. The co-inhibitory molecules on T cell surface are CTLA-4, PD-1, LAG-3, CD160, and TIM-3 [49]. Recently, target immunotherapy using PD-1 and PD-L1 monoclonal antibodies (MoAbs) was demonstrated to significantly induce durable tumor regression and prolong disease stabilization in patients with selected advanced cancers [50]. Krupka et al. indicated that the CD33/CD3 BiTE antibody construct AMG 330 on primary acute myeloid leukemia (AML) cell AMG 330 up-regulates immune checkpoints, PD-1 and PD-L1 on target and effector cells [43]. PD-L1 was strongly up-regulated on primary AML cells upon AMG 330 addition to ex vivo cultures. Through blockade of the PD-1/PD-L1 interaction, AMG 330-mediated lysis, T cell proliferation, and IFNγ secretion were significantly enhanced. Another way of engaging T cells is chimeric antigen receptor–modified T cells (CART). CART uses adoptive transfer of T lymphocytes engineered to express a single-chain fragment variable region (scFv) domain linked to the signaling domain of the T cell receptor (TCR) [51], plus various costimulatory domains. Urbanska et al. combined the application of frBsAbs specific for CD20 or HER2 with T cells that are genetically engineered to express a unique BsAb-binding immune receptor (BsAb-IR) [52]. The lytic activity of primary human T cells expressing the BsAb-IR was specifically redirected against CD20+ leukemic cells or HER2+ epithelial cancer cells. This approach has the unique advantage to simultaneously and locally activate selected population of gene-engineered T cells and trigger the co-stimulatory signals by application of one single bi-specific molecule.

In regards to tumor growth inhibition in vivo, the efficacy of growth inhibition by ds-diabody was much greater than that of its diabody counterpart, although their activity in vitro in cultured cells was similar. This improved antitumor effect in vivo was mainly due to the improved stability of the diabody. The results coincided with those from the previous experiment. So, the ds-diabody was not only useful for malignant cell-lines but also valuable for clinical trials.

In this study, we found that at low doses, Ara-C up-regulated the expressions of CD80, CD86, or both over 100 % in nearly 50 % of specimens of B-ALL patient-derived cells. A combination of diabody or ds-diabody with low-dose Ara-C induced T lymphocytes to exhibit greater cytotoxicity in those B-ALL cells in vitro and in vivo. Furthermore, in vivo, the ds-diabody was more efficient than its parent diabody at inducing tumor cell lysis due to its greater stability. Altogether, our findings illustrated the advantage of combining immunotherapy with chemotherapy in preclinical tumor models, which has set the foundation for developing new clinical strategies aimed at targeting leukemia and lymphoma.

## Conclusion

The therapy of T cells that was induced by diabody or ds-diabody combined with low dose of Ara-C was not only useful for malignant cell-lines but also valuable for clinical trials. In vivo, the ds-diabody was more efficient than its parent diabody due to its greater stability.

## Methods

### Samples and bi-specific antibody

B-ALL cells were obtained from 21 newly diagnosed patients after their informed consent and with the approval of the Clinical Research Ethics Board of the Institute of Hematology, Chinese Academy of Medical Sciences (Tianjin, China). Diagnosis and classification of B-ALL cells were based on the criteria of the FAB group. Cells were maintained in RPMI 1640 (Gibco, Grand Island, NY, USA) containing 10 % fetal bovine serum (FBS) and grown at 37 °C with 5 % CO_2_. Bi-specific anti-CD3 × anti-CD19 diabody and its disulfide-stabilized format (ds-diabody) constructed previously are stored in our lab [31].

### Isolation of PBMC and sorting T lymphocytes

With informed consent, blood samples were collected from healthy volunteers, and peripheral blood mononuclear cells (PBMCs) were isolated by Ficoll-Hypaque density-gradient centrifugation. The interphase cells were washed twice, counted, and tested for viability with trypan blue dye. Then, their monocytes were depleted by adherence to plastic flasks for 2 h. Non-adherent cells were used for T cell isolation using fluorescence-activated cell sorting (FACS). For the sorting of T cells, 1 × 10^8^ PBMC in 1ml phosphate-buffered saline (PBS) was incubated with 15 μl of allophycocyanin (APC)-conjugated anti-human CD2 mAbs (clone RPA-2.10, BD Pharmingen, San Diego, CA, USA), anti-human CD4 mAbs, and anti-human CD8 mAbs mixture at 4 °C for 60 min. After washing twice with PBS, T lymphocytes were sorted using FACS (FACS Aria II Becton, Dickinson and Co., Franklin Lakes, NJ, USA), and the collected cells were cultured in complete RPMI-1640 medium (10 % FBS) supplemented with 50 IU/ml of IL2 for 48 h.

### Co-stimulation molecule expression on B-ALL cells

B-ALL at 1 × 10^6^ cells/ml were incubated with Ara-C at the concentration of 0.25 μM for 72 h. After being washed in PBS twice, the cells were incubated with FITC-conjugated anti-human CD80 mAb (clone L307.4, BD Biosciences, San Jose, CA, USA) and PE-conjugated anti-human CD86 antibody mAb (clone IT2.2, BD Biosciences, San Jose, CA, USA) for 1 h at 4 °C. The stained cells were then analyzed using flow cytometry. The assay was repeated three times for each condition. The B-ALL sample cell-expressed CD80 or CD86 increasing over 100 % were chosen for the following experiments.

### Cytotoxicity test in vitro

Cytolytic activity of T lymphocytes targeted by the diabody and ds-diabody was determined using the calcein-release assay. The T lymphocytes are from one single healthy volunteer. The B-ALL cells stimulated by Ara-C at concentrations of 0.25 μM for 72 h or not were prepared as target cells. For the experiments, quadruplicates of 1 × 10^5^ labeled target cells and CD4+ or CD8+ T cells at different E:T cell ratios ranging from 25:1 to 3:1 per well were added to the round-bottom 96-well plates in a final volume of 100 μl. Diabody and ds-diabody dilutions of 1.0 pM were added to the final volume for the assays. The plates were centrifuged at 250 *×* g for 4 min and incubated for 4 h in a humidified incubator at 37 °C in 5 % CO_2_. After incubation, the cells were concentrated by centrifugation, and the supernatant was transferred to a new 96-well plate. Calcein fluorescence in the supernatant was determined using a fluorescence plate reader (Fluoroskan Ascent FL, Thermo-Fisher, USA; excitation at 485 nm, emission at 535 nm). The percentage of cytotoxicity was calculated using this formula:

$$ \left({\mathrm{F}}_{\mathrm{experimental}\ \mathrm{lysis}}-{\mathrm{F}}_{\mathrm{spontaneous}\ \mathrm{release}}\right)\ /\ \left({\mathrm{F}}_{\mathrm{maximal}\ \mathrm{lysis}} - {\mathrm{F}}_{\mathrm{spontaneous}\ \mathrm{release}}\right) \times 100. $$

Maximal lysis values were obtained by adding 2 % Triton X-100 to labeled target cells. Spontaneous release was observed only in the labeled target cells without T cells and antibodies. Each sample cell assay was repeated four times for each condition.

### Up-regulation of T cell activation markers

To determine the CD4+ or CD8+ T cell surface activation markers, PE-conjugated anti-human CD25 mAb (clone 2A3, BD Biosciences, San Jose, CA, USA) and FITC-conjugated anti-human CD69 mAb (clone L78, BD Biosciences, San Jose, CA, USA) were used as an antibody mixture. Experimental groups were constructed according to a cytotoxicity test in vitro. The ratio of T cells to target cells was 25:1, and the concentration of the diabody or ds-diabody was 1.0 pM. The B-ALL cells that were incubated with Ara-C at concentrations of 0.25 μM for 72 h or not were prepared as target cells. After incubation with the target cells for 4 h, the lymphocytes were collected and washed in PBS with 3 % FBS and 0.1 % NaN_3_. Then, 50 μl of the antibody mixture was added, and the cells were incubated for 1 h at 4 °C. The cells were washed, and the pellets were resuspended in 600 μl of PBS. Samples were analyzed using flow cytometry. The T cells alone and T cells incubating with diabody or ds-diabody without target cells were set up as control.

### Expressions of perforin and granzyme B in activated T cell subpopulation

The ratio of CD4+ or CD8+ T cells to target cells was 25:1, and the concentration of the diabody or ds-diabody was 1.0 pM. The B-ALL cells that were incubated with Ara-C at concentrations of 0.25 μM for 72 h or not were prepared as target cells. Experimental groups were constructed according to a cytotoxicity test in vitro. The cells were washed twice in PBS supplemented with 2 % bovine serum albumin (BSA) and resuspended in 100 μl of fixative (FixPerm kit, Caltag) at room temperature (RT) for 15 min. After further washing, the cell pellet was resuspended in 100 μl of permeabilization solution (FixPerm kit, Caltag) for 15 min at RT and then incubated with a FITC-conjugated anti-perforin mAb (cloned δG9, BD Pharmingen) and PE-conjugated anti-granzyme B mAb (cloned CB9, BD Pharmingen) mixture for 45 min at 4 °C. The cells were then washed twice in PBS-BSA and analyzed by flow cytometry. The T cells alone and T cells incubating with diabody or ds-diabody without target cells were set up as control

### IL2 and IL6 release

IL2 or IL6 concentrations were analyzed in the supernatants of activated CD4+ or CD8+ T cells using a human IL2 or IL6 ELISA kit (NeoBioscience, Shenzhen, China). Experimental groups were constructed according to a cytotoxicity test in vitro. Cytokines released by T cells alone and T cells incubating with diabody or ds-diabody served as control. Approximately 1 × 10^6^ T cells were co-cultured with 4 × 10^4^ target cells. After incubation with the target cells for 4 h, supernatant was removed and analyzed according to the manufacturer’s protocol. The measurements were performed on an ELISA plate reader (Thermo Fisher Scientific, Waltham, MA, USA).

### Release of IL3, TNFα, and IFNγ by activated T cell subpopulation

The ratio of CD4+ or CD8+ T cells to target cells was 25:1, and the concentration of the diabody or ds-diabody was 1.0 pM. T The B-ALL cells that were incubated with Ara-C at concentrations of 0.25 μM for 72 h or not were prepared as target cells. Experimental groups were constructed according to a cytotoxicity test in vitro. RNA isolation, DNase treatment, and RT*-*Total RNA were isolated using TRIzol (Invitrogen), treated with DNase I (Invitrogen), and then 2 μg RNA were reverse-transcribed using Superscript II RT (Invitrogen) following the manufacturer’s instructions in a total volume of 20 μl. Primers for real-time PCR for IL3, TNFα, and IFNγ genes and the housekeeping gene GAPDH were designed using Primer Premier Software 5.0. Human GAPDH primers were 5′-GAAGGTGAAGGTCGGAGT-3′ (forward) and 5′-GAAGATGGTGATGGGATTTC-3′ (reverse). IL3 primers were 5′-GGTTAGCACTGTCTCCAGATCG-3′ (forward) and 5′-TTCTTGCCAGCTCTACCACC-3′ (reverse). IFNγ primers were 5′-GGCTGTTTCTGGCTGTTACTGC-3′ (forward) and 5′-ACTCCTTTTCCGCTTCCTGAGG-3′ (reverse). TNFα primers were 5′-ATGAGCACAGAAAGCATGATCC-3′ (forward) and 5′-ACAAGCAGGAATGAGAAGAGG-3′ (reverse). Real-time PCR was performed with a QuantiTect SYBR Green PCR kit (Takara) on the ABI Prism 7500 Fast Sequence Detection System. Thermal cycling conditions were 95 °C for 15 s, followed by 40 cycles of 5 s at 95 °C and 40 s at 60 °C. PCR reactions were performed in a total volume of 20 μl containing 2 μl of sample cDNA and 0.2 μM of each primer, and the SYBR Green PCR kit was used following manufacturer’s instructions. Each test was amplified in three different wells. The relative quantification of gene expression was assessed by 2^−△△Ct^, △△Ct = △Ct _sample_ − △Ct _control_. Gene expression was assessed by the ratio of the expression level in each patient sample against the mean expression in all normal samples. Also, the quantity of cytokines IL3, TNFα, and IFNγ released into the supernatant by activated T cell subpopulation were measured by ELISA (NeoBioscience, Shenzhen, China) and analyzed according to the manufacturer’s protocol. The T cells alone and T cells incubating with diabody or ds-diabody without target cells were set up as control.

### Tumor growth inhibition in vivo

NOD/SCID mice (Cancer Institute, Chinese Academy of Medical Sciences, Beijing, China) were kept under sterile conditions and received autoclaved food, water, and bedding. Mice that were 6–8 weeks old received 400 cGy from ^137^Cs source 24 h before injection of B-ALL cells. Then, 1 × 10^7^ B-ALL cells were injected into each mouse via the tail vein. Cohorts of six mice were injected with cells from the same B-ALL sample, subjected to the same experimental conditions, and analyzed in parallel. Values shown for engraftment of B-ALL cells in mouse BM are the mean values obtained for all mice that received the same treatment and survived to the time of analysis. Six days after tumor inoculation, each mouse in the same B-ALL sample group was treated with pre-activated T cells (5 × 10^6^ cells/mouse), T cells combined with diabody (2.0 nM/mouse), T cells combined with ds-diabody (2.0 nM/mouse), T cells combined with Ara-C (1 mg/kg), T cells combined with Ara-C and diabody, and T cells combined with Ara-C and ds-diabody via the tail vein. The treatments were administered every 7 days for 3 weeks. Eight weeks after the last treatment, the mice were killed by CO_2_ inhalation, and BM was obtained from the four long bones by flushing with Alpha MEM with 50 % FCS. Cells from mouse tissue were prepared for FACS analysis as described previously [53]. Half the cells were then incubated for 30 min on ice with a mouse IgG1 isotype control (Becton Dickinson Immunocytometry Systems, San Jose, CA, USA), and the other half were incubated with APC mouse anti-human CD45 ( BD Pharmingen; Mouse IgG1, κ) and PE mouse anti-human CD19 ( BD Pharmingen; Mouse IgG1, κ) to detect human cells. Cells were washed and stained with 2 μg/ml propidium iodide. FACS analysis was performed using FACS Aria II. The percentage of CD45^+^ and CD19^+^ cells was determined after excluding 99.9 % of cells labeled with the isotype control and nonviable cells. Nonspecific binding of CD45 on mouse BM cells was reliably ≤0.1 %.

### Statistical analysis

All data were expressed as mean ± standard deviation and statistically analyzed by the Student’s *t* test with SPSS version 18.0 statistical analysis software (IBM, Armonk, NY, USA).

## Abbreviations

APC: Allophycocyanin
Ara-C: Cytarabine
B-ALL: B-acute lymphoblastic leukemia
BiTE: Bi-specific T cell engager
bsAbs: Bi-specific antibodies
CART: Chimeric antigen receptor–modified T cells
CRS: Cytokine release syndrome
ds-diabody: Disulfide-stabilized format
FACS: Fluorescence-activated cell sorting
PBMCs: Peripheral blood mononuclear cells
TAA: Tumor-associated antigen
